# Drug-induced phospholipidosis is not correlated with the inhibition of SARS-CoV-2 - inhibition of SARS-CoV-2 is cell line-specific

**DOI:** 10.3389/fcimb.2023.1100028

**Published:** 2023-08-11

**Authors:** Viktoria Diesendorf, Valeria Roll, Nina Geiger, Sofie Fähr, Helena Obernolte, Katherina Sewald, Jochen Bodem

**Affiliations:** ^1^ Institute for Virology and Immunobiology, University of Würzburg, Würzburg, Germany; ^2^ Fraunhofer Institute for Toxicology and Experimental Medicine ITEM, Member of Fraunhofer International Consortium for Anti-Infective Research (iCAIR), Member of the German Center for Lung Research (DZL), Biomedical Research in Endstage and Obstructive Lung Disease (BREATH), Hannover, Germany

**Keywords:** SARS-CoV-2, phospholipidosis, Vero E6, PCLS, Calu-3, antivirals, Tamoxifen, cell line-specificity

## Abstract

Recently, Tummino et al. reported that 34 compounds, including Chloroquine and Fluoxetine, inhibit SARS-CoV-2 replication by inducing phospholipidosis, although Chloroquine failed to suppress viral replication in Calu-3 cells and patients. In contrast, Fluoxetine represses viral replication in human precision-cut lung slices (PCLS) and Calu-3 cells. Thus, it is unlikely that these compounds have similar mechanisms of action. Here, we analysed a subset of these compounds in the viral replication and phospholipidosis assays using the Calu-3 cells and PCLS as the patient-near system. Trimipramine and Chloroquine induced phospholipidosis but failed to inhibit SARS-CoV-2 replication in Calu-3 cells, which contradicts the reported findings and the proposed mechanism. Fluoxetine, only slightly induced phospholipidosis in Calu-3 cells but reduced viral replication by 2.7 orders of magnitude. Tilorone suppressed viral replication by 1.9 orders of magnitude in Calu-3 cells without causing phospholipidosis. Thus, induction of phospholipidosis is not correlated with the inhibition of SARS-CoV-2, and the compounds act via other mechanisms. However, we show that compounds, such as Amiodarone, Tamoxifen and Tilorone, with antiviral activity on Calu-3 cells, also inhibited viral replication in human PCLS. Our results indicate that antiviral assays against SARS-CoV-2 are cell-line specific. Data from Vero E6 can lead to non-transferable results, underlining the importance of an appropriate cell system for analysing antiviral compounds against SARS-CoV-2. We observed a correlation between the active compounds in Calu-3 cells and PCLS.

## 1 Introduction

During the last 2.5 years, SARS-CoV-2 became a pandemic causing more than 6.6 million deaths worldwide. Although new and effective RNA-based vaccines were developed in 2020 and distributed worldwide, even first-world European countries suffered from rising infections during the summer of 2022 (Dong et al., 2020). Besides the direct effects of the disease, approximately 7% of patients will suffer from long-COVID symptoms in the future, and it became evident that the vaccines will not prevent the viral spread (Cao et al., 2020). Thus, the development of effective viral therapies is essential.

The approaches to developing antiviral therapies can be divided into drug repurposing and developing new antivirals. The first strategy has the obvious advantage that the compounds used are well tested, adverse side effects have been characterised, and the optimal drug concentrations are known. The resulting drugs can either target the virus directly or inhibit specific cellular pathways required for viral replication. However, the mechanism of action needs to be verified in a patient near *in vitro* systems; otherwise, the antiviral effects might not influence patient disease outcomes. The most prominent example of misfired drug-repurposing is Chloroquine, which has been described as a highly active component against SARS-CoV-2 in Vero E6 cells (Wang et al., 2015; Aherfi et al., 2021). Later it was shown that Chloroquine did neither inhibit SARS-CoV-2 in Calu-3 cells nor lead to benefits for infected patients (Hoffmann et al., 2020), indicating that analyses in Vero E6 cells might lead to the identification of suboptimal compounds.

Nevertheless, Tummino et al. published a report in *Science* suggesting that 34 compounds, including Chloroquine and Fluoxetine, inhibit SARS-CoV-2 replication in A549-ACE2^+^ and Vero cells by inducing phospholipidosis (Tummino et al., 2021). This assumption relays on the correlation between phospholipidosis activity and SARS-CoV-2 suppression. However, the data were surprising since contradicting results on Chloroquine had been published before (Hoffmann et al., 2020). Fluoxetine was shown to repress viral replication in human precision-cut lung slices, Vero, Huh-7 and Calu-1 and -3, A459-ACE2^+^ cells and in mouse models (Schloer et al., 2020; Brunotte et al., 2021; Fred et al., 2021; Zimniak et al., 2021; Geiger et al., 2022a; Pericat et al., 2022). Fluoxetine has been shown to repress viral replication by inhibiting the acid sphingomyelinase and acid ceramidase (Geiger et al., 2022a) and influencing the mortality of patients (Hoertel et al., 2022). In this regard, it has been shown that serum C16:0-ceramides are upregulated in infected patients by SARS-CoV-2, promoting a progressive loss of the microvascular barrier function and leading to apoptosis (Petrache et al., 2023), underlining the importance of changes in the sphingomyelinase/ceramidase balance by Fluoxetine for the virus (Kornhuber et al., 2022). However, no one, except Tummino et al., reported a correlation of phospholipidosis with the inhibition of SARS-CoV-2 replication. Thus, it is doubtful that all the compounds described by Tummino et al. use a similar mechanism inhibiting the same target.

## 2 Results and discussion

Since it has been shown for Chloroquine that the outcome of antiviral SARS-CoV-2 assays might depend on the cell line used, we decided to re-analyse a subset of the compounds on the lung adenocarcinoma Calu-3 cell line. First, we determined that our reference concentration of 10 µM did not influence cell metabolism by MTS test as previously described (Zimniak et al., 2021; Friedrich et al., 2022; Geiger et al., 2022a; Geiger et al., 2022b). All compounds did not influence the metabolism at the given concentration.

**Figure 1:**
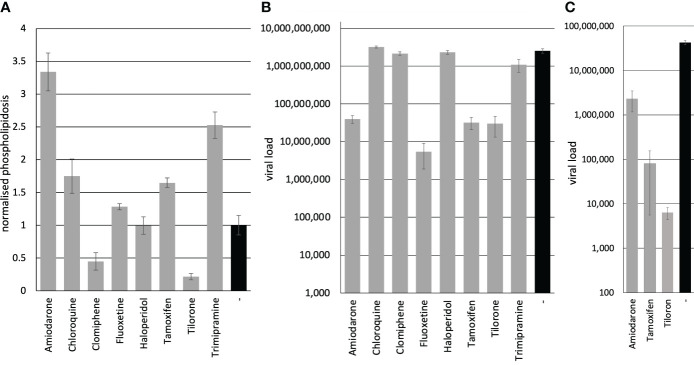
Inhibition of viral replication correlates in Calu-3 cells and human PCLS but is unrelated to phospholipidosis. **(A)** Phospholipidosis activity. Calu-3 cells were incubated with 10 µM of the compounds, and phospholipidosis was analysed after 72 h. **(B)** Inhibition of viral replication. Calu-3 cells were incubated with 10 µM of the compounds and infected with SARS-CoV-2. The viral load was determined after 72 h with RTqPCR. **(C)** Inhibition of SARS-CoV-2 replication on PCLS. PCLS were incubated with 10 µM of the compounds and infected with SARS-CoV-2. Viral infectivity was determined on Vero cells. Bars represent the mean, and error bars the standard deviation of the assays.

We sought to determine the induction of phospholipidosis by the compounds. The phospholipidosis assays were performed on Calu-3 cells, similar to the experiments previously published by Tummino et al., but measured after 72 h simultaneously with genome quantification (Tummino et al., 2021). The Calu-3 cells were incubated with the compounds, and NBD-PE substrate was added 24 h before harvest. The conversion of the substrate was determined in a fluorescence reader. Amiodarone is known to induce phospholipidosis and was used as a positive control (Riva et al., 1987). Amiodarone and Trimipramine increased phospholipidosis by 3.3fold (significance p=1.8x10^-8^) and 2.5fold (significance p=8.6x10^-8^), while Chloroquine (1.7fold) (significance p=0.0002) and Tamoxifen (1.6fold) (significance p=5.4x10^-6^) showed a weaker induction (**Figure 1A**). Fluoxetine (1.3fold) (significance p=0.002), Haloperidol (1fold), Clomiphene (0.4fold) (significance p=9.9x10^-5^), and Tilorone (0.2fold) (significance p=5.4x10^-7^) did not lead to a significant induction (**Figure 1A**; **Table 1**). In contrast, Clomiphene and Tilorone repressed phospholipidosis compared to the solvent control. Next, we analysed the phospholipidosis by Amiodarone and Trimipramine in Vero cells since these compounds showed the highest induction in Calu-3 cells and to ensure that our assays led to comparable results with the previous study. The phospholipidosis was induced 1.7fold by Amiodarone and 2.2fold by Trimipramine in Vero cells. In conclusion, if the induction of phospholipidosis is correlated to the antiviral activity of the compounds, Amiodarone and Trimipramine should suppress viral replication more potent in Calu-3 than in Vero cells and stronger than the other compounds in Calu-3 cells, followed by Chloroquine and Tamoxifen. Furthermore, all other compounds should not suppress viral replication significantly.

**Table 1:** Induction of phospholipidosis is not correlated to the antiviral activity.

Compounds	Phospholipidosis^1^	Log reduction of viral load^2^
Amiodarone	3.3 ± 0.3	1.8
Chloroquine	1.7 ± 0.3	-0.1
Clomiphene	0.4 ± 0.1	0.1
Fluoxetine	1.3 ± 0.0	2.7
Haloperidol	1.0 ± 0.1	0.0
Tamoxifen	1.6 ± 0.1	1.9
Tilorone	0.2 ± 0.0	1.9
Trimipramine	2.5 ± 0.2	0.4
-	1.0 ± 0.1	0.0

^1^normalised to the medium control.

^2^calculated as log_10_(viral load_control_)-log_10_(viral load_sample_).

Next, we analysed the influence of the compounds on viral replication. The viral replication was determined by incubating Calu-3 cells with the compounds and infecting the cells with SARS-CoV-2 (MOI 1). After 24 h, the medium was replaced by a compound-containing medium to remove unbound viruses. All infections were repeated twice. Cellular supernatants were collected three days after infection. Viral RNAs were extracted, and the SARS-CoV-2 RNA genome copy numbers were quantified by RTqPCR. Amiodarone (significance p=2.4x10^-8^), Fluoxetine (significance p=2.1x10^-8^), Tamoxifen (significance p=2.4x10^-8^) and Tilorone (significance p=2.4x10^-8^) reduced viral replication significantly (**Table 1**; **Figure 1B**). In contrast, Chloroquine (significance p=0.005), Clomiphene (significance p=0.07), Haloperidol (significance p=0.35) and Trimipramine (significance p=0.0001) failed to inhibit viral growth in Calu-3 cells (**Figure 1B**). These results confirm the data published for Tilorone on SARS-CoV-2, MERS-CoV (Ekins and Madrid, 2020; Puhl et al., 2021) and Fluoxetine on SARS-CoV-2 (Carpinteiro et al., 2020; Schloer et al., 2020; Zimniak et al., 2021).

Only the treatment with Amiodarone and Tamoxifen showed a correlation between the induction of phospholipidosis and reduced viral replication. However, both compounds reduced viral load similarly, but Tamoxifen induced phospholipidosis by 1.6fold compared to 3.3fold of Amiodarone, which rules out a strict correlation of both (**Table 1**). Furthermore, Trimipramine and Chloroquine induced phospholipidosis but failed to inhibit SARS-CoV-2 replication in Calu-3 cells significantly. Fluoxetine, however, only slightly induced phospholipidosis in Calu-3 cells but reduced viral replication by 2.7 orders of magnitude. Thus, the results for Trimipramine, Chloroquine and Fluoxetine contradict the proposed correlation between antiviral activity and phospholipidosis. Moreover, Tilorone suppressed viral replication by 1.9 orders of magnitude while repressing phospholipidosis in Calu-3 cells. The proposed linear correlation of phospholipidosis and the reduction of viral loads was analysed by calculating the Pearson correlation coefficient with 0.14 and Spearman’s rank correlation p= -0.016878 using the values from **Table 1**. Thus, phospholipidosis is not correlated to the inhibition of SARS-CoV-2 by any of these compounds, making it very likely that the active compound act by other mechanisms.

Finally, we decided to evaluate the SARS-CoV-2 inhibiting compounds Amiodarone, Tamoxifen and Tilorone on Calu-3 cells in patient-near-human precision-cut lung slices. We hypothesised that PCLS represent a preferable model for antiviral substances. Thus, compounds suppressing viral replication *in vivo* should also inhibit viral replication in PCLS. The PCLS were incubated with the compounds and subsequently infected with SARS-CoV-2 at an MOI 10. The medium was exchanged after 24 h, and cellular supernatants were collected after 72 h of infection. The viral load was determined by infecting Vero cells with the supernatants and analyses of viral replication by RTqPCR (**Figure 1C**). All compounds active on Calu-3 cells reduced viral replication in PCLS (significances: Amiodarone p=7.3x10^-6^, Tamoxifen p=4.5x10^-6^, Tilorone p=4.4x10^-6^), showing that Calu-3 cells have a higher predictive level than Vero E6 cells. Furthermore, the previous studies on Chloroquine and our report on Fluoxetine support this hypothesis that Vero E6 cells might not represent a preferential assay system for the analyses of compounds against SARS-CoV-2.

In summary, we provide evidence that the selection of an inappropriate cell system for antiviral assays, such as Vero E6 or A549-ACE2 cells, might lead to the choice of compounds inactive in patients with Chloroquine as a prominent example. Furthermore, we identify Amiodarone, Tamoxifen and Tilorone as potential antivirals active in human PCLS.

## 3 Methods

### 3.1 Cellular toxicity assays

Cell toxicity was determined by analysing the cellular metabolism with the CellTiter 96^®^ AQueous One Solution Cell Proliferation Assay (Promega, Waldorf, Germany). The cells were seeded into a 96-well plate. The compounds were added, and the cells were incubated for 72 h. Then 10 µl of the MTS solution (Promega, Waldorf, Germany) was added to the medium, and the cells were further incubated for 1 h. Finally, the absorption was determined and compared to the untreated control. The assays were performed in six replicates, and the standard deviation was calculated.

### 3.2 Determination of viral genome copies

The virus isolate has been described before (Schmidt et al., 2021; Zimniak et al., 2021). The genome copy number was determined by RTqPCR 72 h after infection. Calu-3 cells were incubated with the compounds and subsequently infected with SARS-CoV-2, as described before (Zimniak et al., 2021; Friedrich et al., 2022; Geiger et al., 2022a; Geiger et al., 2022b). The medium was exchanged to remove defective viruses after 24 hours with the medium containing the compounds. After 72 h, 200 µl of the medium was collected, and viral genomes were purified with the High Pure Viral Nucleic Acid kit (Roche, Mannheim, Germany). SARS-CoV-2 RNA genomes were quantified with the dual-target SARS-CoV-2 RdRP RTqPCR assay kit, which contains universal SARS-CoV-2 primers, and with viral RNA multiplex master kit (Roche) with a LightCycler 480 II (Roche). The provided standard was used for genome copy-number quantification using the LightCycler 480 II Software (Roche).

### 3.3 Phospholipidosis assays

Calu-3 cells were seeded in an optical black 96-well plate with a clear bottom (Greiner, Frickenhausen,

Germany) at a density of 20000 cells per well. On the next day, 10 μM of compounds were added. After 24 h, the medium was replaced, and after 48 h, 7.5 μM NBD-PE substrate (Merck, Darmstadt, Germany) was added. The fluorescence was measured after 72 h with an excitation wavelength of 463 nm and emission at 536 nm with an Ensight plate reader (PerkinElmer, Rodgau, Germany). The significance was calculated by the student’s t-tests. The Pearson correlation coefficient was calculated with the Jupyter-Notebook software.

### 3.4 Human precision-cut lung slices

The human lung lobes were acquired from patients undergoing lobe resection for cancer at Hannover Medical School. The use of the tissue for research was approved by the ethics committee of the

Hannover Medical School and complies with the Code of Ethics of the World Medical Association (number 2701–2015). Infection of human precision-cut lung slices and determining viral infections were performed as described before (Zimniak et al., 2021; Geiger et al., 2022a). The human PCLS were incubated for 1 h at 37°C in DMEM/F12 medium (Life Technologies, Darmstadt, Germany) supplemented with 1% Penicillin/Streptomycin (Lonza, Verviers, Belgium) and separated on a 48-well dish. The compounds were added, and the PCLS were infected with SARS-CoV-2 with a high MOI of approximately 10. Viral infectivity was determined by infecting Vero cells with 100 µl of the viral supernatants for 3 days.

## Data availability statement

The original contributions presented in the study are included in the article/supplementary materials. Further inquiries can be directed to the corresponding author.

## Ethics statement

Human lung lobes were acquired from patients undergoing lobe resection for cancer at Hannover Medical School. The use of the tissue for research was approved by the ethics committee of the Hannover Medical School and complies with the Code of Ethics of the World Medical Association (number 2701–2015).

## Author contributions

VD, VR, and NG contributed equally to this work and share the first authorship. VD, VR, NG, SF, and HO performed the experiments. KS and JB supervised the project. JB wrote the manuscript. All authors contributed to the article and approved the submitted version.
